# Group personality, rather than acoustic noise, causes variation in group decision-making in guppy shoals

**DOI:** 10.1038/s41598-025-03225-y

**Published:** 2025-05-29

**Authors:** Molly A. Clark, Ella Waples, Andrew N. Radford, Stephen D. Simpson, Christos C. Ioannou

**Affiliations:** 1 School of Biological Sciences, University of Bristol, Bristol, BS8 1 TQ UK; 2 School of Natural Sciences, Macquarie University, Sydney, NSW 2109 Australia

**Keywords:** Anthropogenic noise, Collective behaviour, Leadership, *Poecilia reticulata*, Social behaviour, White noise, Animal behaviour, Behavioural ecology, Freshwater ecology, Ecology, Zoology

## Abstract

Group living has essential fitness benefits for many species. While numerous studies have explored how environmental conditions impact collective movement, their impact on decisions made in a social context—a central component of group-living—is poorly documented. In this study, we assess how acoustic noise impacts group decision-making, cohesion and activity in fish shoals, using Trinidadian guppies (*Poecilia reticulata*) as a model species. Movements within a radially symmetric five-armed maze were measured using high-resolution trajectory data from video-tracking software. The behaviour of groups with and without continuous acoustic white noise were measured over a four-day testing period in a repeated-measures design. We found no significant change in swimming speed or group cohesion with additional acoustic noise. However, there was evidence for fewer following events (moves into already occupied arms) in the noise treatment compared to the control, but this additional noise had no effect on leadership attempts (moves into empty arms). We found strong evidence for consistent, repeatable differences between groups in all behavioural parameters indicating strong personality variation at the group level. Rather than environmental factors, these results provide evidence that consistent group-level differences dominate variation in collective behaviour, including group decision-making, in fish shoals.

**Supplementary Information:**

The online version contains supplementary material available at 10.1038/s41598-025-03225-y.

## Introduction

Group living in animals can improve foraging, efficiency of movement and the avoidance of predators, with important fitness benefits^1,2^. Many of these benefits are obtained through cohesive movement mediated by social interactions and self-organisation that allow for information transfer between individuals^3^. Group-living also has costs^1^, and whether or not conformity is favoured by group members to maintain cohesion can depend on individual fitness requirements^4^.

Some of the benefits of group-living are provided by collective decision-making which can enhance abilities to exploit resources and evade predation^3^. Group decisions can involve choices such as the direction of travel, where to forage, or where to nest^5,6^. Group decisions may be egalitarian, where decisions are equally shared among individuals, or involve leadership by a few individuals within a group, or even by a single individual^5^. Leadership is dependent on the ‘willingness’ of other individuals to follow, thus leadership attempts may be successful or unsuccessful depending on the actions of others in the group^7^. These leadership-followership dynamics are essential to group functioning, as they determine which information is shared and acted upon. This influences the ability of groups to coordinate and make adaptive responses to their environment^5^.

Fish in shoals are thought to use cues based on the location and movement of other individuals, rather than active signals, to mediate collective movement^8,9^. In this way, collective motion enables collective decision-making in the absence of direct communication. Leaders emerge through positional changes within a group, with those at the front of a shoal having more influence over the direction of movement^10,11^. While studies of the mechanisms of collective decision-making are extensive, the impact of ecological context is less well known. Evidence that group decision-making can change with ecological conditions has been demonstrated in Trinidadian guppy (*Poecilia reticulata*) shoals, where Ioannou et al.^12^ found that group decision-making differed depending on whether individuals were caught from a low- or high-predation river. Guppies from high predation populations showed stronger differentiation into leader and follower roles, marked by a strong negative correlation between the number of leadership attempts and following events, compared to guppies from environments with lower predation pressure^12^. The majority of studies testing how environmental stressors affect collective behaviour in fish shoals focus on collective motion, rather than group decision-making^13^, which is often the outcome of collective motion and leads to important fitness-related decisions. Therefore, there have been relatively few empirical studies of how abiotic conditions affect the dynamics of group decision-making. Chamberlain and Ioannou^14^ found that with experimentally induced water turbidity, three-spined sticklebacks (*Gasterosteus aculeatus*) became more independent in their decision making, shifting their behaviour away from that of the group, due to the visual constraints imposed by turbidity.

Anthropogenic noise pollution is a growing concern, particularly in aquatic environments^15,16^. Human activities including ship traffic, construction, tourism and recreation have increased significantly over the past century, and with them so have environmental sound levels^17^. Due to the relatively high density of water compared with air, noise travels further and faster underwater, meaning the impacts of noise pollution can be more widespread^15,16^. Animals use acoustic signals for communication and gather additional acoustic information about their environment. Anthropogenic noise can thus mask auditory reception of important stimuli, decreasing the signal-to-noise ratio^15^. Additionally, noise can distract individuals from important tasks, such as foraging^18,19^, and cause direct physiological stress^20,21^. Environmental noise can disrupt animal behaviour in many ways, and its relevance has grown with the increasing prevalence of anthropogenic sources.

Impacts of acoustic noise pollution can have knock-on effects on social behaviour^13^. Cetaceans have been found to alter their calls, in frequency^22^ and duration^23^, as a result of boat noise, demonstrating that acoustic noise can cause marine mammals to modify their acoustic communication. Noise-induced distraction and stress can also disrupt collective behaviour by impairing the effective use of information through cross-modal effects^24^. Noise has been shown to impact collective movement negatively by reducing the coordination and cohesion of shoals^25,26^. However, whether noise disrupts the decision-making processes of groups has yet to be addressed.

We aimed to investigate how acoustic noise impacts group-level behaviours in shoals of Trinidadian guppies, including group cohesion, movement dynamics and collective decision-making. Similarly to Ioannou et al.^12^., we recorded groups of guppies swimming in a five-arm radially symmetrical maze whilst experiencing an acoustic playback of either a recording from their housing tank (i.e. a control treatment) or this same recording with white noise overlaid to simulate acoustic noise pollution. By using high-resolution tracking data obtained by overhead video footage, we explored how group-wide behaviours (speed, exploration, cohesion, and collective decision-making) of fish shoals changed with noise disturbance. To assess the consistency of behavioural responses we used a repeated-measures design, where the same groups of fish were tested across multiple days in both treatments. This approach allowed us to test whether changes in behaviour due to noise disturbance were consistent over time, and to explore group-level personality traits. Consistent behavioural responses across repeated trials would be evidence of group-level personality^27–30^. Such consistent group-level differences in fish shoals have been demonstrated to occur across ecological contexts^29^. Thus, including a measure of group-personality in our design can build on previous work and provide useful information on how group personality is influenced by anthropogenic disturbance.

We hypothesised that additional acoustic noise would reduce overall group cohesion and exploration behaviour, based on prior studies showing that noise can impair coordination and reduce cohesion in fish shoals through masking or distraction effects^25,26^. Previous studies have shown that over repeated trials, animal groups tend to acclimate to experimental arenas, leading to reduced cohesion and altered exploratory behaviour^30–32^. Therefore, we also expected group cohesion to reduce over the days of testing, while group speed and group exploration may increase, as the environment becomes more familiar. In terms of collective decision-making, we expected that added noise would affect leadership dynamics, with a smaller proportion of leadership attempts being successful due to fewer following events. This could result from distraction or stress making potential followers less responsive to social cues.

## Materials and methods

### Animal ethics statement

The experiment was carried out at the University of Bristol aquarium and was approved by the university’s Animal Welfare and Ethical Review Body (UIN/17/060 and UIN/17/075). Methods followed ASAB Guidelines for the treatment of animals in behavioural research and are reported in accordance with ARRIVE guidelines. Individuals were exposed only to two 20-minute additional-noise treatments to minimise stress. After the experiment, fish were monitored to ensure they were healthy, and housed in preparation for future experiments.

### Study species

Guppies were originally collected from a high-predation site on the Guanapo river in Trinidad, West Indies, in April 2019. They were exported to the John Krebs Field Station, University of Oxford, and reared for a minimum of three generations with a controlled breeding plan to prevent inbreeding and ensure genetic diversity was maintained. Individuals from the third generation were transported by car for approximately 1.5 h in sealed plastic bags, with 1 third water and 2 thirds oxygen, within insulated containers and then housed at the University of Bristol aquarium in December 2020, 9 months prior to experimental trials. The precise ages of individuals at the time of the experiment were unknown. All 72 individuals were kept in one 90 L glass tank (70 × 40 × 35 cm L x W x H) which contained approximately 100 to 150 individuals total. The tank contained artificial plants, plastic tunnels and a slow bubbling air stone. Water temperature was maintained at 25 ± 1^o^C (mean ± SD). Lighting was maintained on a 12:12 h light: dark cycle. Fish were fed on a diet of live and fresh food (frozen blood worms, cyclops, mysis, brineshrimp and live banana worms) and ZM Granular pellets (© Copyright 2021 ZM Fish Food and Equipment).

### Experimental design

**Fig. 1:**
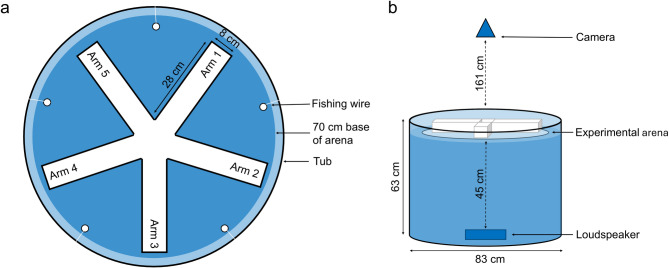
The experimental set up. **a** Overhead view of the experimental arena suspended in the tub by fishing wire. **b** Side view of experimental arena in tub with camera and loudspeaker placement. Not to scale.

The experimental arena consisted of a five-arm maze constructed out of matt white corrugated PVC. Each arm (28 × 8 × 18 cm, L x W x H) was attached with white electrical tape to a 70 cm diameter circular base made of white acrylic. The experimental arena was suspended in a 83 × 63 cm (D x H) cylindrical tub with transparent 8 lb fishing wire attached to the base at five points to reduce vibrations (Fig. 1a). A Sony AX53 digital 4 K video camera recorder was suspended over the arena 161 cm above the base of the experimental arena (Fig. 1b). The experimental arena was shielded from direct lighting and potential disturbance by white cotton sheets suspended around the arena and tub, while a black cotton sheet between the overhead fluorescent lighting and the camera reduced glare and reflections. The water level in the experimental arena was kept constant at 7.5 cm. Two Hepo HP-608 300 W Aquarium heaters set to 25^o^C, one in the five-arm maze and one in the wider tub, and one Aquarium Systems Duetto 50 4 W filter, were kept on between trials. The experimental arena was refilled every day before trials were started but not between trials, and 50% of the water in the tub was replaced weekly. Water was allowed to fill gaps in the corrugated plastic walls of the experimental arena to prevent air pockets insulating sound passing through.

### Noise treatments

An ambient playback track of sound recorded from the guppies’ housing tank was used as the control treatment, while the noise treatment consisted of white noise overlaid onto this ambient playback track (similar to^33^). Only one track was used for each treatment (one for added white noise and one for the ambient control), as computer-generated standardised white noise exhibits minimal variation between tracks. White noise is characteristically consistent, and any minor variation is expected to be negligible, making it unlikely to introduce substantial variability between trials. The ambient playback track was recorded with a HiTech HTI-96-MIN omnidirectional hydrophone with inbuilt preamplifier and a Zoom H1n digital recorder (manufacturer-calibrated sensitivity: −164.3 dB re 1 V/mPa; frequency range 20e30 000 Hz, High Tech Inc., Gulfport, MS, U.S.A.) connected to a Zoom H6 digital recorder (PCMM10, 48 kHz sampling rate, Sony Corporation, Tokyo, Japan) in the housing tank at half water depth. A 5-minute recording was looped to generate a 30-minute track of ambient sound for the control treatment. The continuous white noise treatment was generated in Audacity (version 2.4.2) and overlaid onto the ambient sound track to simulate the effect of added noise on to their normal ambient soundscape.

Sounds recordings were made in both the housing tank and the experimental arena. Sound pressure recordings were made with a HiTech HTI-96-MIN omnidirectional hydrophone with inbuilt preamplifier connected to a Zoom H6 digital recorder. Reported sound levels are averages taken from recordings in each area of the experimental arena; the level was higher in the middle of the experimental arena because the small size of the arena caused the noise to concentrate more in the middle during playback (see Supplementary Table S1 and Figure S1 for more detailed analysis). The recorded RMS sound-pressure level in the guppies’ housing tank was 107 ± 2 dB re 1 µPa (mean ± SD), which the ambient control mimicked; the white noise treatment was distinctly louder at 135 ± 3 dB re 1 µPa. To measure the particle motion in the experimental arena, accelerometer recordings were taken using a M20-40 Geospectrum Technologies Inc. accelerometer and Zoom H6 digital recorder (see Supplementary Figure S2 for more detailed analysis). All acoustic recordings were analysed in MATLAB 2013a using paPAM^34^. A bandpass filter was applied between 100 and 2000 Hz to account for the most common hearing sensitivities in fish^35^. All heaters and filters were switched off during recordings, and water depth and temperature were kept constant, matching experimental arena conditions used during the trials.

Both playback tracks were 30 min in duration to provide continuous sound during the trial period. They were played from a SanDisk Clip Jam MP3 player through a DNH Aqua-30 underwater loudspeaker (frequency response 100–10,000 Hz, ADD DNH details) connected to an amplifier (Kemo Electronic GmbH; 18 W; frequency response: 40–20 000 Hz) and a Maplin 12 V 12 Ah battery. The loudspeaker was suspended in a plastic box using elastic to minimise vibrations and this was submerged in the bottom of the 63 × 83 cm black tub, 45 cm below the experimental arena (Fig. 1b).

### Experimental protocol

At the start of each week, groups of four female guppies (29.5 ± 2.4 mm, mean ± SD standard body length, *n* = 72 individuals) were formed from individuals haphazardly caught from the housing tank 24 h before the start of the experimental trials. Individuals were randomly assigned to one of ten groups by shuffling the group numbers (from 1 to 10) randomly. Each fish was caught one at a time using a hand net and assigned to the corresponding group in the randomised order. The group numbers were then reshuffled, and the process repeated until four fish were in each group. Groups were formed in this way to minimise potential variation between groups, for example if bolder fish are caught first^36,37^. Each group was held in a fry net (12.5 × 16 × 13.5 cm, L x W x H), with two nets per 45 L tank (70 × 20 × 35 cm, L x W x H), in the same room as their original housing tank. Fish remained in these groups throughout the testing period of four days. Fish were fed ZM Granular pellets (© Copyright 2021 ZM Fish Food and Equipment) in the fry nets after being put into groups, and at the end of each day of trials. To control for differences in social and reproductive behaviour between males and females, only single-sex female groups were tested^38,39^.

All trials took place between 0900 and 1500 from the 20th September to 5th November 2021. Groups were tested once a day over four consecutive days in a repeated-measured design. The treatment that a group received alternated from one day to the next so all groups were tested twice in each treatment (noise or control), where a treatment (added noise) trial one day would be followed by a control trial the next day, and vice versa. Treatment order was decided by random shuffling of group numbers on the first day of testing: the first half of the random list were given the control treatment first and the second half given the noise treatment first. The testing order of groups within a day was also randomised. All randomisations were done using R (version 4.1.2^40^).

At the start of a trial, all heaters and filters were turned off (pilot tests were conducted to ensure water temperature remained constant throughout the test day), the camera was switched on, and the appropriate playback track was started, to play through the underwater loudspeaker. A group of four fish were transferred to the experimental arena by a hand net and given a three-minute acclimation period inside a 12 (diameter) x 30 cm clear cylinder made of 5 mm rigid acrylic in the middle of the arena. At the end of the acclimation period, the cylinder was carefully lifted out of the arena with a clear rod attached to the cylinder with fishing wire to minimise disturbing the fish. After the 15-minute trial period, the camera filming was stopped, and the fish were caught and placed back into their fry net (unless it was the final day of testing, when they were released into a new housing tank for fish already used in the experiment). The heaters and filters were turned on for the period between the trials, and the experimental arena was set up for the next trial.

### Video analysis and data processing

Video files were recorded in 4K (3840 × 2160 pixel) resolution with a frame rate of 25 fps. The automated tracking software idTracker^41^ was run in MATLAB 2014a to obtain the trajectories of individual fish during the trials at each video frame. Identities were maintained within each trial but could not be confirmed across trials of the same group. Trajectories were then processed in R (version 4.1.2^40^). In cases where there were missing coordinates for any individual in a frame, all data were removed from that frame. This is because with missing information for any individual, social parameters cannot be reliably calculated. Trajectories were smoothed using a Savitsky-Golay filter using the *Trajr* package in R (version 1.3.0^42^). Additionally, when the speed of an individual exceeded 25 pixels per frame, all data points for that frame were removed as this speed was assumed to be a result of erroneous tracking; cumulative density plots were used to determine the threshold at which high speeds were considered erroneous and removed. All trials were cropped to 13.5 min each, removing the first 1.5 min of the trial where the fish would often remain still after removal of the acclimation tube. Overall, tracked video footage for 72 trials across 18 groups was obtained.

### Behavioural parameters

Using the processed trajectory data, the mean speed of each individual across each trial (pixels/frame) was calculated, and then the mean of these mean speeds across the four fish was calculated. Which arm each fish was in at every frame, or whether they were in the middle area joining the arms (Fig. 1a), was determined using the coordinates of the corners of each arm, with these coordinates measured using ImageJ version 1.53^43^. From this, four behavioural parameters were calculated per trial: mean cohesion (the standard deviation of the number of fish in each arm at each frame, averaged [mean] across all frames); the number of leadership attempts (moves into an empty arm); the number of following events (moves into an arm already occupied by another individual); and the total number of moves made into each arm (the sum of the leadership attempts and following events). When calculating the number of leadership attempts and the number of following events four trials were excluded due to high movement classifictation error. This error was defined by the proportion of inter-arm transitions that did not pass through the mazes central junction, an indiciation of potentially misclassified movements. Trials with more than 40% of such transitions were excluded (threshold decided by Figure S3), leaving the sample size for leadership and followership analysis at 68 trials from 18 groups.

### Statistical analysis

Mean speed, mean cohesion, and the total number of moves into arms (i.e. exploration) were analysed as response variables in linear mixed models (LMMs; constructed using the lmer function *lme4*, version 1.1.30^44^). To analyse the proportion of moves into arms that were leadership attempts rather than following events, the number of leadership attempts and following events in each trial were combined into a two-column matrix using the cbind function in R (e.g. cbind(number of leadership attempts, number of following events)). This was used as the response variable in binomial generalised linear mixed models (GLMMs; constructed using the glmer function *lme4*, version 1.1.30^44^). Based on the results from these models of the proportion of moves into arms that were leadership attempts, additional LMMs were constructed with the number of leadership attempts and the number of following events as separate response variables.

All models included group identity as a random effect and the time of day the trial began (trial start time) as a covariate. The inclusion of sound treatment (additional noise or ambient control) and day of testing (1 to 4) was varied between models to test for their independent and combined effects. For each response variable (i.e. mean speed, group cohesion, exploration, proportion of leadership attempts to following events, number of leadership attempts, number of following events), five models were constructed which included either the treatment*day interaction, treatment and day as main effects only, treatment only, day only, or neither of these terms as a null model. To determine which explanatory variables were important for explaining variation in the response, the Akaike Information Criterion values corrected for small samples sizes (AICc) were compared for each model using the ICtab function in R (bbmle version 1.0.24^45^. The model with a ΔAICc of zero is the most likely given the data. Models with a ΔAICc of greater than two units less than the null model were considered to have strong support, and therefore the fixed effects of the model were considered important in predicting the response variable^46,47^. For each fixed effect in the best-fitting models, we report the estimate, standard error, and 95% confidence interval.

To complement the cohesion index, a probability distribution function (PDF) was calculated to describe the spatial distribution of the fish within the arena. For each frame, the number of unique arms (including the central junction) occupied by at least one fish was counted. As there were four fish per trial, the number of occupied arms could range from 1 (all fish in the same arm or zone) to 4 (each fish in a separate arm or zone). For each trial, the proportion of frames in which 1, 2, 3, or 4 arms were occupied was calculated, providing a probability distribution of group dispersion states. The means and standard deviation across all trials per treatment group (control and noise) were reported, allowing for comparison of the typical distribution of fish within the arena across experimental conditions.

Individual identities were not tracked across trials, therefore repeatability was assessed at the group level only. Consistent group-level repeatability was tested using the *rpt* function in the *rptR* package (version 0.9.22^48^) to calculate the intraclass correlation coefficient (ICC) for the best-fitting models. Repeatability quantifies the proportion of total variance in a response variable that can be attributed to consistent differences between groups, relative to the total variance (including residual variance). Bootstrap resampling (1000 iterations) was applied to compute 95% confidence intervals for the repeatability estimates. We report the repeatability (R), 95% confidence intervals (CIs) and p-values from likelihood ratio tests (LRTs), with *p* < 0.05 indicating significant repeatability. The binomial GLMM for the proportion of leadership attempts to following events could not be used to calculate ICC or LRT values due to convergence and boundary fit issues, which is a known limitation when estimating ICC for binomial models^49^.

All analyses were conducted using R (version 4.1.2^40^) in RStudio^40,50^. Multicollinearity was tested in all models by calculating the variation inflation factors (VIFs), with no strong evidence of multicollinearity found (VIF < 3 in all cases). The assumption of normality in the residuals was tested in all LMMs using QQ plots, and residuals were plotted against fitted values to ensure homogeneity of variance. Maximum likelihood (ML) was used to fit all LMMs, rather than the *lme4* default restricted maximum likelihood (REML), because models within the comparisons contained different fixed effects^51,52^. Under- or over-dispersion in all binomial GLMMs was tested using the residual diagnostics for mixed regression models (DHARMa^53^).

## Results

**Fig. 2:**
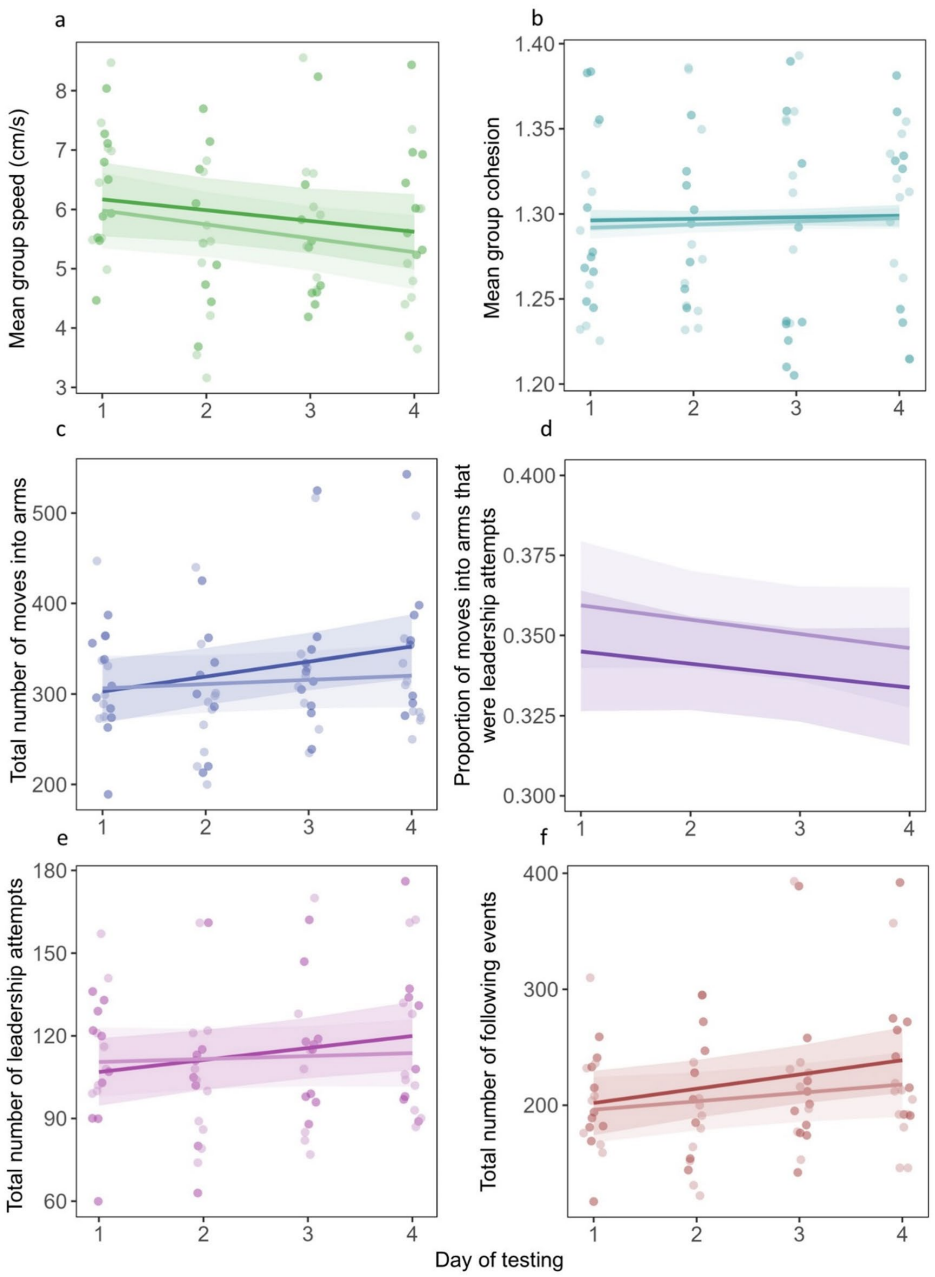
The relationship between the day of testing in both noise (lighter colour) and control (darker colour) treatments and the: **a** mean speed of individuals; **b** mean group cohesion (standard deviation of the number of fish in the same arm at any time); **c** total number of movements into arms during a trial; **d** proportion of moves into arms that were leadership events; **e** number of leadership attempts; and **f** number of following events. Fitted lines are calculated from fixed-effect estimates from the model with all main effects and the treatment*day interaction term. Shaded areas are 95% confidence intervals. Points are individual data points (**a**, **b** and **c**: 18 groups, 72 trials; **d**, **e** and **f**: 18 groups, 68 trials) and are jittered (0.1 units) to reduce overplotting. The plot was generated using the *sjplot* package in r^54^.

For mean speed, the model with testing day as the only explanatory variable was within 0.3 AICc units of the most likely model (with both sound treatment and day as main effects) and thus has similar predictive power. Including treatment did not substantially make the model more likely, suggesting limited evidence for an effect of treatment on mean speed (treatment vs. control: estimate = − 0.30 ± 0.18 SE, 95% CI [–0.66, 0.06]; Table 1(1), Fig. 2a). All models with day as a term were more likely (by more than 2 AICc units) than the null model, with the mean speed of individuals decreasing over the days of data collection (Fig. 2a).

When testing the variables that predicted the cohesion of fish during the trials, the null model had the strongest support, and the inclusion of treatment or testing day did not substantially improve model likelihoods (Table 1(2)). This indicates weak evidence for effects of treatment or day on group cohesion (Fig. 2b). These results are supported by the PDF (Table S2) which shows no significant difference in the way fish moved throughout the arena between the control and treatment groups. The PDF revealed that fish most often occupy one (42% control, 36% treatment) or two (44% control, 48% treatment) arms at a time, suggesting that individuals were generally not alone. Occupation of four arms simultaneously (i.e., each fish occupying a separate arm) occurred very rarely (1% in both treatment and control).

The analysis of exploration behaviour (total number of moves made into arms) suggested that including testing day as a main effect made the models more likely, as only models including day were substantially more likely than the null model, and the models that included treatment did not further improve the likelihood by more than 0.5 AICc units (Table 1(3)). The model containing only treatment had a higher AICc value than the null model, providing little support for a treatment effect on the exploratory behaviour of groups. The best-supported model indicated that groups became more exploratory across testing days (estimate = 10.61 ± 3.69 SE, 95% CI [3.25, 17.97]), while there was limited evidence that treatment affected exploration (treatment vs. control estimate = − 14.22 ± 8.22 SE, 95% CI [–30.64, 2.19]; Fig. 2c).

When analysing the proportion of moves into arms that were leadership attempts, the best-supported model had treatment as the only explanatory variable (Table 1(4)). This model was 1.9 AICc units less than the null model, indicating modest support^46^ for an effect of treatment on the proportion of leadership attempts. The estimated effect of treatment (noise vs. control) was 0.06 ± 0.03 SE (95% CI [0.002, 0.11]) on the logit scale, suggesting a higher proportion of leadership attempts in the noise treatment (Fig. 2d). Including day as an additional predictor did not improve the model likelihood (AICc = 0.3 difference).

Given that in the previous analysis the difference in AICc values (1.9) was close to the threshold of 2 units that would indicate strong evidence that the proportion of leadership attempts was higher in the noise treatment, we explored this further by separately analysing the number of leadership attempts and following events. When the number of leadership attempts was the response variable, the most likely model was that including only day as an additional fixed effect. However, this was only 1.3 AICc units lower than the null model, indicating limited evidence that testing day influenced the number of leadership attempts (estimate = 2.70 ± 1.39 SE, 95% CI [–0.08, 5.48]; Table 1(5), Fig. 2d). All other models had AICc values higher than the null model and were therefore not supported.

For the number of following events as the response variable, the most likely model included both treatment and day as main effects (Table 1(6)). The model without treatment, including the day and start time only, was 1.5 units from the most likely model, suggesting modest support for a treatment effect on the number of following events (treatment vs. control estimate = 13.38 ± 6.69 SE, 95% CI [–26.72, − 0.03]). Inclusion of testing day in the models improved their likelihood by > 2 AICc units, suggesting strong evidence for day in predicting the number of following events (estimate = 9.9 ± 3.00 SE, 95% CI [3.82, 15.79]). The total number of following events increased over the days of testing and was higher in the control treatment compared to the noise treatment (Fig. 2e).

**Table 1:** The ∆AICc for models explaining variation in the: (1) mean speed of individuals, (2) mean group cohesion, (3) group exploration (the total number of moves individuals made into arms), (4) proportion of moves into arms that were leadership attempts, (5) number of leadership attempts, and (6) number of following events.

	(1)			(2)		
Response variable	Mean speed			Group cohesion		
		∆AICc	d.f.		∆AICc	d.f.
Explanatory variables	Treatment + day + start time	0	6	Start time only (null model)	0	4
	Day + start time	0.3	5	Treatment + start time	0.5	5
	Treatment * day + start time	2.4	7	Day + start time	0.7	5
	Treatment + start time	5.3	5	Treatment + day + start time	1.1	6
	Start time only (null model)	5.7	4	Treatment * day + start time	3.3	7

	(3)			(4)		
Response variable	Group exploration			Proportion of leadership attempts		
		∆AICc	d.f.		∆AICc	d.f.
Explanatory variables	Treatment + day + start time	0	6	Treatment + start time	0	4
	Treatment * day + start time	0.3	7	Treatment + day + start time	0.3	5
	Day + start time	0.5	5	Start time only (null model)	1.9	3
	Start time (null model)	5.1	4	Day + start time	2.2	4
	Treatment + start time	5.3	5	Treatment * day + start time	2.7	6

	(5)			(6)		
Response variable	Number of leadership attempts			Number of following events		
		∆AICc	d.f.		∆AICc	d.f.
Explanatory variables	Day + start time	0	5	Treatment + day + start time	0	6
	Start time only (null model)	1.3	4	Day + start time	1.5	5
	Treatment + day + start time	2.2	6	Treatment * day + start time	1.9	7
	Treatment * day + start time	3.5	7	Treatment + start time	7.4	5
	Treatment + start time	3.5	5	Start time only (null model)	7.8	4

The null model includes start time as the only fixed effect. All models include group identity as the random effect. Treatment is noise or control, day is the day of testing (1 to 4).

### Repeatability

**Fig. 3:**
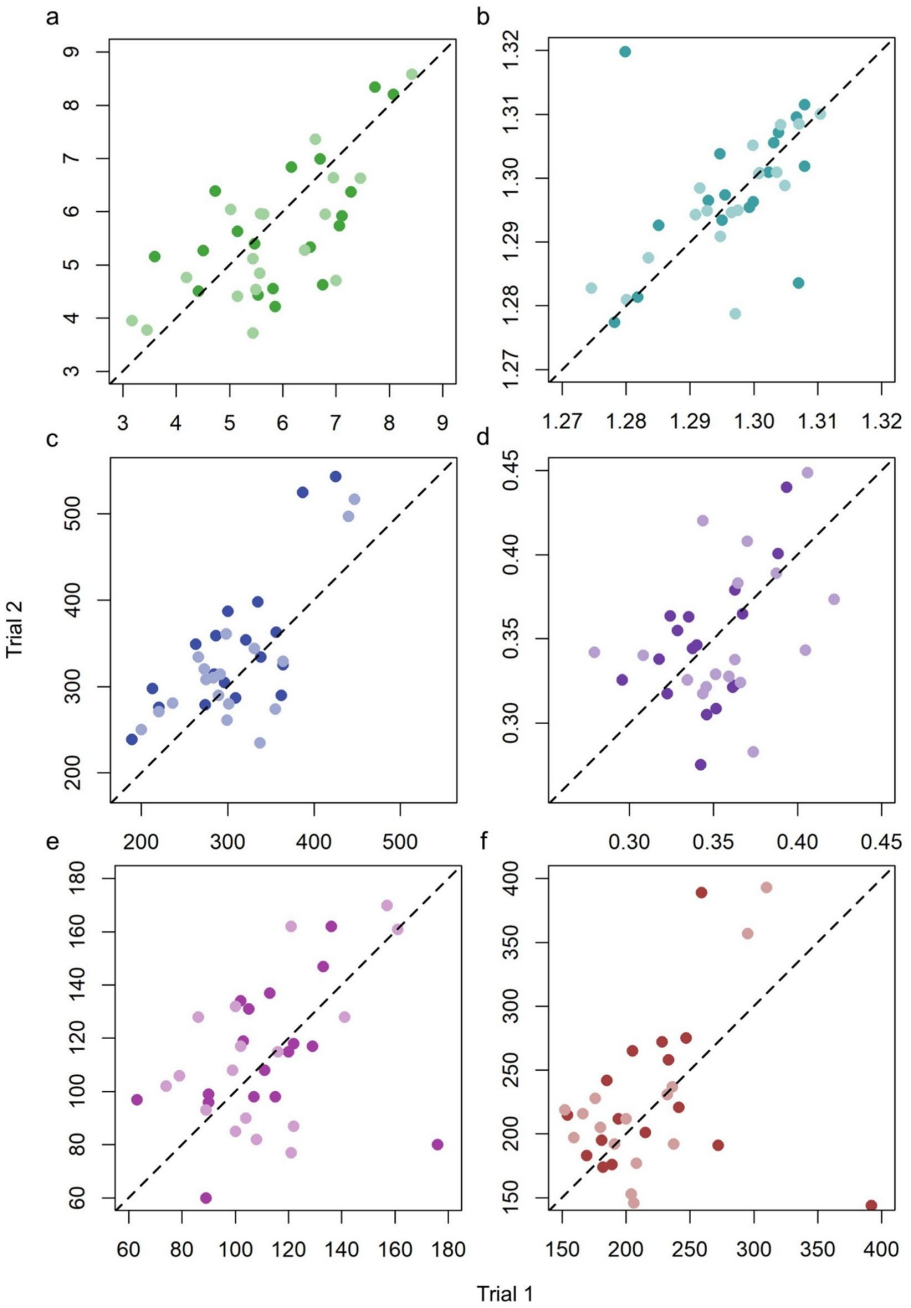
Relationships between response variables in the first and second control trials (darker points) and first and second treatment trials (lighter points) for each response variable: **a** mean speed of individuals (pixels per frame); **b** mean group cohesion (standard deviation of the number of fish in the same arm at any time); **c** total number of movements into arms during a trial; **d** proportion of moves into arms that were leadership events; **e** number of leadership attempts; and **f** number of following events. The dashed line is x = y.

For each response variable, the ICCs provided strong evidence for consistent behavioural differences between groups (Table 2; Fig. 3). A substantial proportion of the variation in behaviours (i.e. for mean speed, group cohesion, total moves into arms, leadership attempts and following events) was attributable to group identity, as indicated by high repeatability values. For instance, mean speed showed a repeatability of 0.676, indicating that 68% of the total variance in speed was attributable to differences between groups. Similarly, high repeatability values were observed for group cohesion (*R* = 0.714), exploration (*R* = 0.744), and following events (*R* = 0.776). All behaviours showed highly significant p-values (all < 0.001) supporting the conclusion that repeatability is significantly greater than zero.

**Table 2:** Repeatability (R) and confidence intervals (CIs) from the intraclass correlation coefficient (ICC), and p-values from likelihood ratio tests (LRTs) for all best-fit models with group identity as the random effect.

Model	Random effect	d.f.	*R*	95% CI	*p*-value
Mean speed ~ treatment + day + start time	Group ID	7	0.676	0.456, 0.824	< 0.001
Group cohesion ~ start time	Group ID	7	0.714	0.471, 0.844	< 0.001
Total moves into arms ~ treatment + day + start time	Group ID	7	0.744	0.525, 0.871	< 0.001
Prop. leadership attempts ~ treatment + start time	Group ID	6	NA	NA	NA
Total leadership attempts ~ day + start time	Group ID	6	0.706	0.451, 0.844	< 0.001
Total following events ~ treatment + day + start time	Group ID	6	0.776	0.575, 0.889	< 0.001

All models include the fixed effect of start time, additional terms May include treatment (noise or control) and/or day of testing (1 to 4). The binomial GLMM for the proportion of leadership attempts could not be used to calculate ICC or LRT values.

## Discussion

The behaviour of animal groups under anthropogenic disturbances, such as noise pollution, is a growing field of study. Previous research has explored how shoaling behaviour is affected by various environmental factors, including temperature^55^, turbidity^14,56^, dissolved oxygen^57^, dissolved carbon dioxide^58^, darkness^59^ and different combinations of multiple stressors^32,33,60^. Our results suggest that guppies may be more resilient to added acoustic noise than expected from previous studies^13,61,62^ as neither swimming speed nor group cohesion were affected by added white noise. There was some effect of white noise on the number of following events, as guppies in the noise treatment exhibited fewer following events than those in the control group. The most pronounced source of variation, however, was differences between groups, which showed high degrees of repeatability.

The absence of change in group cohesion as a result of increased acoustic noise was unexpected, as previous studies demonstrate that anthropogenic noise can reduce cohesion and coordination in shoals of juvenile seabass^26^, and bluefin tuna (*Thunnus thynnus*) in the field^25^. Conversely, Eurasian minnows (*Phoxinus phoxinus*) were found to be more cohesive when exposed to added continuous sound^61,62^. These studies used recordings of anthropogenic noise sources rather than white noise, which may yield different responses as a result of stress^19^. However, when white noise has been used in laboratory experiments, we observe similar results as found in other species, for example in three-spine sticklebacks (*Gasterosteus aculeatus*^33^), which showed that group cohesion was not impacted by the white noise treatment. Furthermore, in our study, group cohesion did not change over the days of testing. In studies of collective movement, experiments often find that over repeated trials, group cohesion reduces due to acclimation to experimental arenas^30,31^. This is unlikely to be due to differences in the fish species used, as Allibhai et al.^32^. also used guppies and demonstrated this acclimation effect on group cohesion. Instead, the arena design may account for these differences, as our 5-arm radial maze was unlike the open arenas used in previous work^30–32^.

There was some evidence that the proportion of all moves that were leadership events was higher in the noise treatment than in the control, with no change over the days of testing. Further examination revealed that this was because there were fewer following events in the noise treatment compared to the control, with no change in the number of leadership attempts. Added acoustic noise may be distracting individuals within a group from detecting social cues and following others^18,19^, although an effect of stress due to the added noise would be predicted to increase the tendency of fish to follow others^63^. The movements of other fish in the group are critical for collective movement and decision-making^8,9^, and are a form of social information; our results suggest that noise could be restricting the ability of group members to use social information, and causing them to be unresponsive to leadership cues. This could have consequences for the fitness benefits provided by group-living, where the use of social information is key to making better decisions. However, this reduction in followership did not have a detectable impact on group cohesion as this was not affected by added acoustic noise, thus the fitness effects of reduced followership deserves further study.

The most pronounced source of variation in the swimming speed and collective behaviour variables was the consistent differences among the groups tested over multiple days. These consistent, repeatable differences among groups across all behavioural parameters is considered group-level personality variation, as found in previous research^27–30^. This group-level personality variation may arise from stable individual differences within the group or emerge from interactions between group members^64^. High variability in group personality could influence how susceptible or resilient a group is to external disturbances, suggesting that personality traits might buffer groups from potential stressors^65^. However, with only two replicates per group per treatment and only two levels (control and noise treatment), our study was not designed to explore this, but this would likely be a fruitful avenue for future research.

Our results reflect recent findings on a major predator of guppies, pike cichlids. In the study of Brown and Ioannou^66^, feeding motivation, and hence the risk they likely pose to their prey, of the pike cichlid *Saxatilia proteus* was found to be unchanged by environmental parameters (temperature and light), but showed strong among individual variation that was consistent over time. The importance of personality variation in the risk that pike cichlids pose has also been demonstrated in the natural habitat of guppies^67^. Together with our current study, the next step is to explore the interaction between the personality types of individual pike cichlids and personality types of small guppy shoals, for example testing whether the success of the predators or prey is dependent more on the personality type of one than the other, or is dependent on the interaction between the personality types of both predator and prey.

Anthropogenic noise is increasingly recognised as a significant disturbance in aquatic ecosystems, where masking, distraction and stress generate unimodal or cross-modal effects on animal behaviour^15,16^. In group-living species, these disturbances may destabilise group structure and reduce cohesion, both of which are vital for maintaining the benefits provided by group-living, such as improved predator avoidance^1,2^. Our study examined how decision-making in guppy shoals responds to added white noise and found that, overall, guppies were robust to this environmental stressor in the behaviours examined. However, as the laboratory environment already had relatively high ambient noise levels, it is possible that the guppies had developed some degree of habituation to noise prior to the experimental trials. Different forms of white noise, such as impulsive or irregular sounds, or replicating anthropogenic stimuli like boat engine recordings, may elicit different behavioural responses, and highlights an interesting direction for future research. A subtle reduction in following behaviour in the noise treatment suggests that white noise may disrupt social decision-making processes. This finding underscores the importance of examining specific social behaviours, like decision-making. Understanding these nuances will be crucial as we further explore how environmental stressors influence group-living species and their complex social structures.

## Electronic supplementary material

Below is the link to the electronic supplementary material.

Supplementary README

Supplementary Data 1

Supplementary Script 1

Supplementary Material 1

## Data Availability

All data generated or analysed during this study are included in this published article and its supplementary information files (Supplementary Data 1). The analysis code is provided as a script (Supplementary Script 1), accompanied by a README.
