# Supplementary material for: Group personality, rather than acoustic noise, causes variation in group decision-making in guppy shoals
Source: Sci Rep. 2025 May 29;15:18801. doi: 10.1038/s41598-025-03225-y (PMC12122675; doi:10.1038/s41598-025-03225-y)
Supplement: Supplementary file 4 — Supplementary Material 1 [file 41598_2025_3225_MOESM4_ESM.pdf]

Supplementary material for

Group personality, rather than acoustic noise, causes variation in group decision-making in guppy shoals

Molly A. Clark, Ella Waples, Andrew N. Radford, Stephen D. Simpson & Christos C. Ioannou

This file includes:

Supplementary Tables S1 and S2
Supplementary Figures S1 to S3

Table S1 | The RMS noise level in dB re 1 μPa for each area of the experimental arena reported as mean ± standard deviation. Hydrophone recordings were taken from the experimental arena for both treatments using a HiTech HTI-96-MIN hydrophone and Zoom H1n digital recorder (sensitivity levels: 3 for ambient playback, 5 for white noise playback). 10-second recordings were taken at the end, middle, and start of each arm, and one recording taken in the middle of the experimental arena. S1 refers to the corresponding letters in Figure S1. Hydrophone recordings were analysed in MATLAB v2013a using paPAM (Nedelec *et al.,* 2016).

S1	Area of experimental arena	Ambient control treatment (RMS level in dB re 1 μPa)	Added white noise treatment (RMS level in dB re 1 μPa)
A	Middle of arena	112	138
B	Start of arms	107 ± 0.9	136 ± 1.0
C	Middle of arms	106 ± 1.2	135 ± 1.0
D	End of arms	105 ± 0.6	132 ± 0.7

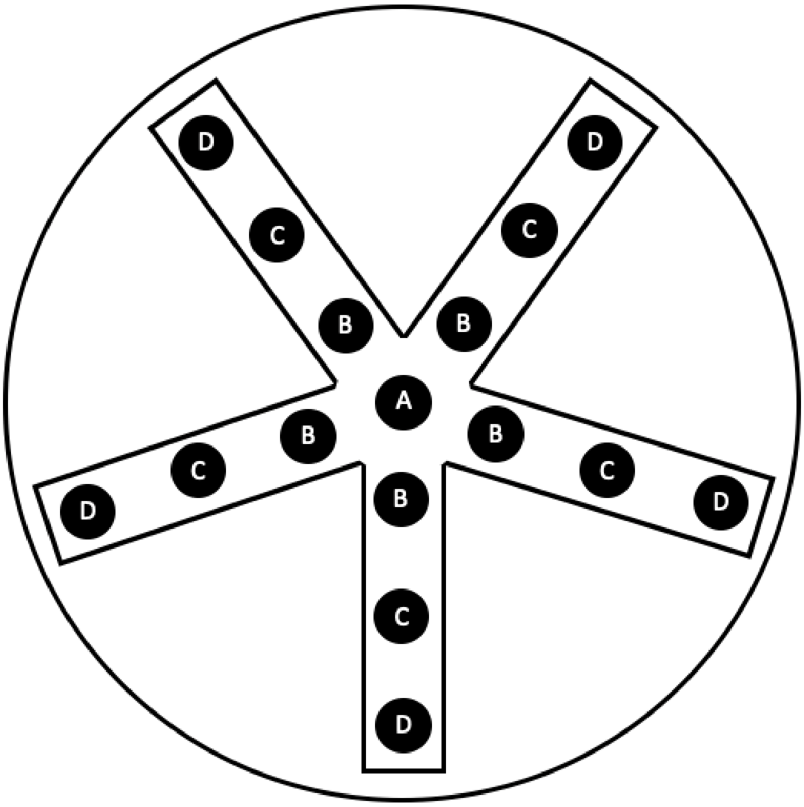

Figure S1 | Areas of experimental arena where hydrophone recordings were taken. Corresponding to data in Table S1.

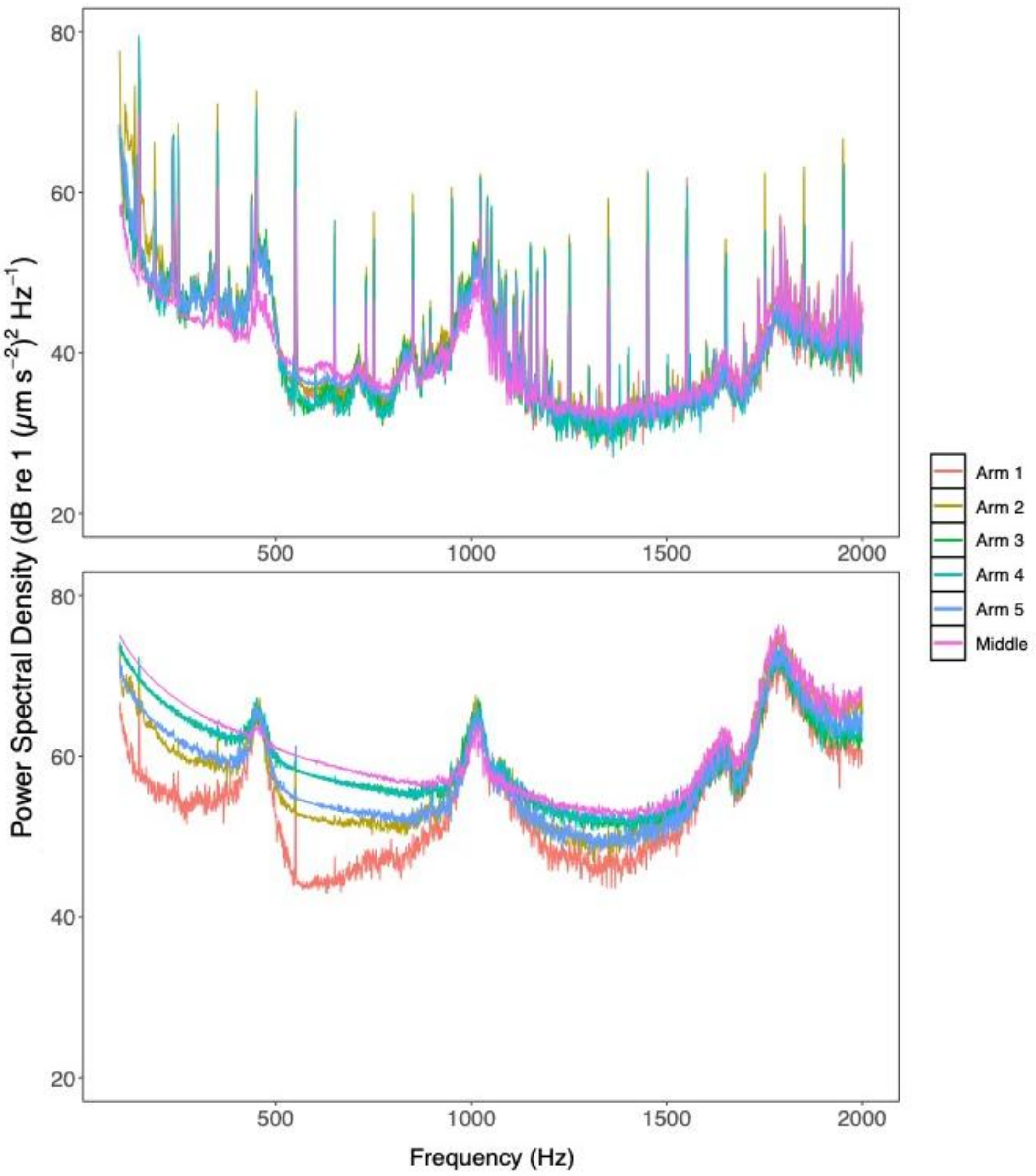

Figure S2 | Power spectral density plot for particle motion in each area of the arena for both the ambient control treatment (top) and white noise treatment (bottom). Recordings were taken using a M20-40 Geospectrum Technologies Inc. accelerometer and Zoom H6 digital recorder (sensitivity level 3 for ambient, 5 for white noise playback). Recordings were made in middle of the experimental arena and once in each arm. Due to the shallow water depth and relatively large size of the accelerometer, it had to be orientated horizontally and thus could only record on the Z axis. 30-second recordings were taken and these were cropped to 10 seconds for analysis. The mean values for particle motion throughout the experimental arena were 30.58 – 81.56 dB re 1 µm s^{-2} in the ambient control treatment and 49.66 – 97.99 dB re 1 µm s−2 in the added white noise treatment.

Table S2 | Probability distribution function (PDF) showing the number of arms (including the central junction) occupied by at least one fish per frame, ranging from 1 (all fish together) to 4 (each fish in a different location). Mean and standard deviation (SD) are reported for control and treatment, calculated from all trials.

Number of occupied arms at each frame	Control		Treatment	
	Mean	SD	Mean	SD
1	0.42	0.11	0.36	0.12
2	0.44	0.07	0.48	0.07
3	0.13	0.05	0.15	0.06
4	0.01	0.01	0.01	0.01

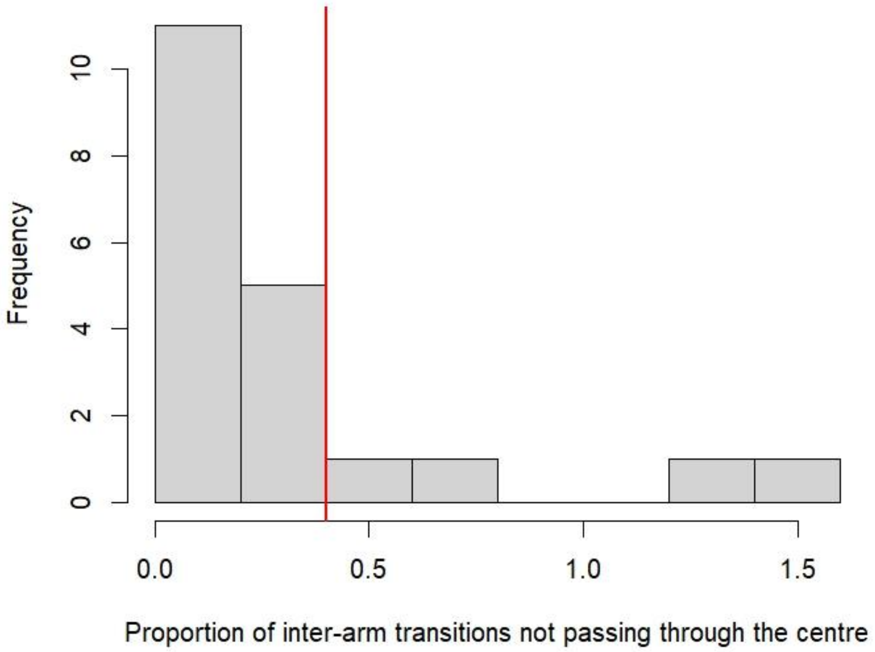

Figure S3 | Histogram representing the criteria for the exclusion of trials from the analysis of leadership attempts and following events. The red line indicates the exclusion threshold: trials with more than 40% of inter-arm transitions not passing through the central junction of the maze were excluded from leadership/followership analyses. Frequency represents the number of trials. Four outlier trials were excluded.
